# Skeletal-related events and mortality among men diagnosed with advanced prostate cancer: The impact of alternative measures of radiation to the bone

**DOI:** 10.1371/journal.pone.0175956

**Published:** 2017-04-18

**Authors:** Eberechukwu Onukwugha, Young Kwok, Jay P. Ciezki, Candice Yong, Catherine Plaisant, Chandana A. Reddy, C. Daniel Mullins, Brian Seal, Adriana Valderrama, Arif Hussain

**Affiliations:** 1 University of Maryland School of Pharmacy, Baltimore, MD, United States of America; 2 University of Maryland School of Medicine, Baltimore, MD, United States of America; 3 Cleveland Clinic Radiation Oncology, Cleveland, OH, United States of America; 4 University of Maryland College Park, College Park, MD, United States of America; 5 Bayer HealthCare, Whippany, New Jersey, United States of America; 6 Veterans Affairs Medical Center, Baltimore, MD, United States of America; University of L'Aquila, ITALY

## Abstract

**Purpose/Objective(s):**

Skeletal-related events (SREs), which include radiation to the bone (RtB), can occur among patients with bone metastasis (BM). There is a recognized potential for misclassification of RtB when using claims data. We compared alternative measures of RtB to better understand their impact on SRE prevalence and SRE-related mortality.

**Methods and materials:**

We analyzed data for stage IV prostate cancer (PCa) cases identified between 2005 and 2009 in the Surveillance, Epidemiology, and End Results registry linked with Medicare claims. We created two measures of RtB: 1) a literature-based measure requiring the presence of a prior claim with a BM code; 2) a new measure requiring either that the BM code coincided with the radiation episode or that the duration of the radiation episode was less than or equal to 4 weeks. We estimated adjusted hazard ratios of an SRE using both measures among stratified samples: no metastasis (M0), metastasis to bone (M1b) and other sites (M1c).

**Results:**

The study sample included 5,074 men with stage IV PCa (median age 77 years), of whom 22% had M0, 54% had M1b, and 24% had M1c disease at time of PCa diagnosis. Based on Approaches 1 and 2, the proportion with probable RtB was 5% and 8% among M0, 30% and 30% among M1b, and 25% and 27% among M1c patients. Among M0 patients, the adjusted hazard ratio (AHR) associated with an SRE was 1.27 when using Approach 1 (95% confidence interval, CI: 0.95–1.7) and 1.49 when using Approach 2 (95% CI: 1.14–1.96). However, the impact of SREs on mortality did not differ between both approaches among M1b and M1c patients.

**Conclusion:**

We found that alternative measures used to define RtB as SRE in claims data impact conclusions regarding the effect of SREs on mortality among M0 but not M1 patients.

## Introduction

The 5-year survival for localized and regional prostate cancer (PCa) is 100%, but only 28% for men with metastatic disease.[1] In PCa, bone is the most common site of metastasis and affects up to 8 in 10 men with metastatic disease.[2] Patients with bone metastasis (BM) are at risk of developing skeletal complications known as skeletal-related events (SREs), which typically include pathological fractures, spinal cord compression, bone surgery and palliative radiation to the bone (RtB). SREs have been reported to be associated with impaired health-related quality of life, increased morbidity, and substantial economic burden.[3–6] A number of studies have also shown that SREs negatively affect survival [7, 8] and that the impact on survival varies by the type of SRE.[9] As a cluster of events, SREs occur in approximately 50–60% of patients with BM secondary to PCa, with RtB commonly reported as the most prevalent SRE subtype followed by pathological fractures.[10]

The estimated prevalence of each SRE subtype has varied across studies due to differences in study populations and methodology. RtB has been reported to occur in 60–90% of PCa patients with BM.[5, 7, 8] Although population-based studies that utilize administrative claims data offer the advantage of large sample sizes and evidence that reflects real-world clinical practice, they are often limited by the lack of specific clinical information such as laboratory test results or disease progression. In claims-based observational studies that examine RtB, procedural codes are used to identify RtB. Generally, patients are first identified to have BM using diagnosis codes, and all radiation episodes that occur on or after claims-based evidence of BM are considered RtB. A drawback of this approach is that claims-based measures of BM based on diagnosis codes have limited validity for identifying patients with BM. Further, the procedural codes for radiation do not specify the site of radiation [11] and hence cannot differentiate between palliative radiation therapy to the bone and radiation therapy to the prostate gland or other anatomic sites. As a result, counting all radiation episodes as RtB could result in an overestimation of RtB since some patients who only received radiation targeted to other sites would be misclassified as having received RtB.

There is currently no validated algorithm for identifying RtB using claims data. To our knowledge, there is limited evidence regarding the implications of various decisions made during the development of claims-based measures for identifying RtB. As current technological developments facilitate the availability of larger, linked datasets, it will become increasingly important to understand the implications of fundamental decisions that are made when creating variables for analysis using claims data. National Comprehensive Cancer Network guidelines recommend 8 to 9 weeks of radiation therapy to the prostate gland.[12] In a previous study conducted on patients with BM secondary to pancreatic cancer, the median duration for radiation to the bone was 15 days.[13] Based on the practice of administering shorter duration of radiation therapy for palliation to the bone compared to treatment to the prostate gland, we sought to determine if the length of radiation therapy may be informative for identifying RtB using claims data. The objective of this proof-of-concept study was to investigate the prevalence of SREs and the impact of SREs on mortality across alternative claims-based measures for identifying RtB.

## Materials and methods

### Study population

This was a retrospective cohort analysis of linked Surveillance, Epidemiology, and End Results (SEER) cancer registry and Medicare claims data on men aged 66 years or older and diagnosed with incident American Joint Committee on Cancer Tumor-Node-Metastasis (AJCC-TNM) stage IV prostate cancer between 2005 and 2009. The additional inclusion criteria were: 1) continuous enrollment in Medicare Parts A and B during the 12 months prior to and including the month of diagnosis; 2) not enrolled in a Medicare Advantage plan during the 12 months pre-diagnosis period; 3) no history of other cancers within 5 years prior to PCa diagnosis; 4) at least 30 days of follow-up; and 5) non-missing information from SEER data regarding the site of metastasis at diagnosis. Using collaborative stage information from SEER, patients were categorized into mutually exclusive groups as follows: 1) no distant metastasis (M0); 2) metastasis to the bone (M1b); and 3) metastasis to other sites (M1c). Men with metastasis to distant lymph node only (M1a) were excluded as the small sample size (N = 204) did not permit separate analysis; and their different prognosis did not justify grouping them with the M0, M1b, or M1c groups.

### Measures

The outcome of interest was all-cause mortality. The primary independent variable was evidence of any skeletal-related event (SRE) occurrence as identified from inpatient, carrier, and outpatient claims using the relevant diagnosis and procedure codes. The referent group was men who did not experience any SRE following PCa diagnosis. Similar to the definition of SREs in clinical trials conducted in men with bone metastasis secondary to PCa [14–16], SREs identified in this study included four SRE subtypes: fractures, spinal cord compression, bone surgery, or radiation to the bone (RtB). RtB included external beam radiation therapy, radiopharmaceutical therapy, intensity modulated radiotherapy and stereotactic radiosurgery.

Given the lack of a validated measure for identifying RtB in administrative claims, we used two measures to identify RtB in this study and compared the mortality impact of SREs using both measures. The only difference between the SRE measures was the definition for RtB. Approach 1 for identifying RtB was based on prior literature.[17] Approach 2 was developed based on more stringent use of the BM diagnosis codes in claims data and clinical input regarding shorter length of RtB compared to radiation to the prostate gland. Previous findings using the EventFlow[18] data visualization software suggested that the length of radiation therapy may be useful in distinguishing between RtB and radiation to the prostate gland during the time period covered by this SEER Medicare dataset.[19] The length/duration of radiation episode was determined by consolidating consecutive radiation claims with a gap of fewer than 7 days. In Approach 1, RtB was identified by any radiation claim that occurred after a claim with a bone metastasis (BM) ICD-9 diagnosis code of 198.5 (secondary malignant neoplasm of bone and bone marrow). In Approach 2, RtB was identified based on a BM diagnosis code directly coinciding with the period of the radiation episode *OR* the duration of the radiation episode was less than or equal to 4 weeks. We selected a 4-week maximum duration because the duration of radiation therapy targeting the bone tends to be shorter than the duration of radiation to the prostate tissue, where radiation to the bone ranges between 1–30 days and radiation to the prostate tissue spans 7 to 8 weeks.[13, 20]

### Statistical analysis

Cox proportional hazards (PH) regression models were used to examine the association between SREs and all-cause mortality across the two measures for identifying SREs. Interaction terms between the SRE indicator and the binary indicators for sites of metastasis at diagnosis were statistically significant, indicating that the mortality impact of SREs depended on the initial site of metastasis. Hence, we examined the mortality impact of SREs in the M0, M1b, and M1c subpopulations.

The regression models included the following potentially confounding patient-level baseline variables: demographic factors (age, race, marital status, urban residence), clinical factors (tumor differentiation), at least 1 month of state buy-in, region of SEER registry, and year of diagnosis. Claims data from the 12 months prior to diagnosis were used to obtain other potentially confounding baseline measures: Charlson Comorbidity Index, presence of osteoporosis, poor performance status (measured by an indicator for any use of skilled nursing facility, hospitalization, walking aids, wheelchairs, or home oxygen), and the use of preventive services (measured by an indicator for any bone mineral density test, flu vaccination, prostate-specific antigen test, or colorectal cancer screening).

Adjusted survival curves were produced for the full sample and stratified samples to examine the SRE effect graphically.[21] We reported time-invariant adjusted hazard ratios (HRs) when the PH assumption was not violated, and time-specific HRs in the case of statistically significant non-proportional hazards associated with SREs. Survival models were also estimated with PCa-specific mortality and non-PCa mortality. The cut-off value for statistical significance was 0.05. All statistical analysis was conducted using Version 9.3 of the Statistical Analysis Software System. This study was approved by the University of Maryland Baltimore Institutional Review Board.

## Results

The study sample included 5,074 men with stage IV PCa, of whom 22% had M0, 54% had M1b, and 24% had M1c disease at the time of PCa diagnosis. The median age for the sample was 77 years. Table 1 shows other sample characteristics, stratified by metastasis status at diagnosis. Compared to the M0 sample, the M1b and M1c samples were generally older, had a higher proportion of non-Hispanic African Americans, and had greater comorbidity burden as measured by Charlson Comorbidity Index (Table 1). All-cause mortality and PCa-specific mortality in the full sample were 55% and 31% at a median (mean; min; max) follow up of 579 days (652; 30; 1826). Of the 5,074 men, the proportion who had any fracture, SCC, and BS was 23.5%, 6.4%, and 5.9%. As shown in Table 1, the prevalence of each SRE subtype varied by site of metastasis at diagnosis.

**Table 1:** Descriptive statistics for patients diagnosed with stage IV prostate cancer in 2005–2009 (N = 5,074).

Variables	Full Sample (N = 5,074)		M0 Sample (N = 1,117)		M1b Sample (N = 2,726)		M1c Sample (N = 1,231)		P-value
	N	Col %	N	Col %	N	Col %	N	Col %	
Skeletal-related events									
Fracture	1,194	23.5	161	14.4	726	26.6	307	24.9	<0.01
Spinal cord compression (SCC)	322	6.4	35	3.1	194	7.1	93	7.6	<0.01
Bone surgery (Surg)	301	5.9	35	3.1	186	6.8	80	6.5	<0.01
Age									<0.01
66–69	954	18.8	407	36.4	364	13.4	183	14.9	
70–74	1,064	21.0	312	27.9	533	19.6	219	17.8	
75–79	996	19.6	188	16.8	573	21.0	235	19.1	
80–84	992	19.6	103	9.2	595	21.8	294	23.9	
85+	1,068	21.1	107	9.6	661	24.3	300	24.4	
Race									0.01
Non-Hispanic White	3,881	76.5	882	79.0	2,091	76.7	908	73.8	
Non-Hispanic African American	627	12.4	104	9.3	349	12.8	174	14.1	
Hispanic	315	6.2	76	6.8	151	5.5	88	7.2	
Other	251	5.0	55	4.9	135	5.0	61	5.0	
Married	3,090	60.9	786	70.4	1,612	59.1	692	56.2	<0.01
Urban residence	4,502	88.7	1,002	89.7	2,412	88.5	1,088	88.4	0.50
Poorly Differentiated Tumor	3,356	66.1	958	85.8	1,738	63.8	660	53.6	<0.01
Charlson Comorbidity Index									<0.01
Zero	2,787	54.9	698	62.5	1,443	52.9	646	52.5	
One	991	19.5	230	20.6	508	18.6	253	20.6	
Two or higher	864	17.0	141	12.6	505	18.5	218	17.7	
Missing	432	8.5	48	4.3	270	9.9	114	9.3	
Osteoporosis	49	1.0	NR	<1.0	28	1.0	NR	<1.0	0.89
Poor performance status proxy*	1,279	25.2	204	18.3	723	26.5	352	28.6	<0.01
Use of preventive services**	3,448	68.0	895	80.1	1,761	64.6	792	64.3	<0.01
State buy-in (at least 1 month)	718	14.2	106	9.5	412	15.1	200	16.3	<0.01
Region of SEER registry									0.02
Northeast	975	19.2	204	18.3	506	18.6	265	21.5	
South	948	18.7	207	18.5	493	18.1	248	20.2	
Midwest	720	14.2	140	12.5	416	15.3	164	13.3	
West	2,431	47.9	566	50.7	1,311	48.1	554	45.0	
Year of diagnosis									0.04
2005	1,098	21.6	232	20.8	584	21.4	282	22.9	
2006	1,028	20.3	197	17.6	559	20.5	272	22.1	
2007	976	19.2	241	21.6	498	18.3	237	19.3	
2008	1,004	19.8	229	20.5	547	20.1	228	18.5	
2009	968	19.1	218	19.5	538	19.7	212	17.2	

NR: not reported due to small cell size (<11), per data use agreement.

*Poor performance status proxy in the 1 year prior to diagnosis was measured by an indicator for any use of SNF, hospitalization, walking aids, wheelchairs, or home oxygen.

**Preventive services in the 1 year prior to diagnosis was measured by an indicator for any of the following: bone mineral density test, flu vaccination, prostate-specific antigen test, or colorectal cancer screening.

The proportion who received any radiation therapy in the full sample was 36%. Based on Approaches 1 and 2, the proportion with probable RtB was 23.0% and 24.3%, respectively (Table 2). The proportion with any SRE was 40.1% and 41.4%, respectively, for Approaches 1 and 2. The prevalence of probable RtB and any SRE was highest in the M1b population and lowest in the M0 population. The median times from PCa diagnosis to the first fracture, SCC, and bone surgery were 146, 322, and 173 days respectively. The median times to the first radiation episode based on Approaches 1 and 2 were 162 days and 145 days, respectively.

**Table 2:** Proportion of patients with palliative radiation to the bone (top) and any skeletal-related event (bottom) across measures*.

Radiation to the bone**		
	Approach 1	Approach 2
Stage IV (N = 5,074)	23.0%	24.3%
M0 (n = 1,117)	4.9%	8.0%
M1b (n = 2,726)	29.5%	30.0%
M1c (n = 1,231)	25.1%	26.7%
Any skeletal-related event***		
	Approach 1	Approach 2
Stage IV (N = 5,074)	40.1%	41.4%
M0 (n = 1,117)	19.8%	22.7%
M1b (n = 2,726)	47.3%	47.8%
M1c (n = 1,231)	42.6%	44.0%

*The 2 measures for measuring radiation to the bone were: 1) Radiation claim occurred concurrent with or after a claim with a bone metastasis (BM) code; 2) Either BM code directly coincided with the period of the radiation episode or the duration of the radiation episode was less than or equal to 4 weeks.

** Radiation to the bone included external beam radiation therapy, radiopharmaceutical therapy, intensity modulated radiotherapy and stereotactic radiosurgery.

***Patients were considered to have a skeletal-related event if they experienced any radiation to the bone, fracture, spinal cord compression, or bone surgery. The only difference between Approach 1 and 2 was the definition for probable radiation to the bone.

Table 3 compares the distribution for episode duration among radiation episodes with and without BM diagnosis code. For radiation episodes with BM code present, the majority of radiation episodes (>70%) were less than or equal to four weeks in duration. For radiation episodes without a BM code, more than half of the radiation episodes in the M1b and M1c populations were less than or equal to four weeks in duration. In contrast, among the M0 population, just 11.2% of the radiation episodes without BM code was less than or equal to four weeks in duration; while 47.8% of the radiation episodes were greater than eight weeks in duration.

**Table 3:** Distribution for duration of radiation episodes, by presence of bone metastasis (BM) diagnosis code during radiation episode.

	Stage IV Sample		M0 Sample		M1b Sample		M1c Sample	
	(N with radiation episodes = 1,806)		(N with radiation episodes = 492)		(N with radiation episodes = 936)		(N with radiation episodes = 378)	
Duration of radiation episode	No BM code	BM code	No BM code	BM code	No BM code	BM code	No BM code	BM code
	n = 843	n = 963	n = 454	n = 38	n = 269	n = 667	n = 120	n = 258
0–4 weeks	32.3%	>80%	11.2%	>70%	55.8%	87.1%	59.2%	85.3%
>4–6 weeks	12.3%	>5%	10.6%	NR	13.8%	8.7%	15.8%	8.9%
>6–8 weeks	21.1%	<5%	30.4%	NR	8.9%	2.6%	13.3%	NR
>8 weeks	34.3%	<5%	47.8%	NR	21.6%	1.7%	11.7%	NR

**Fig 1:**
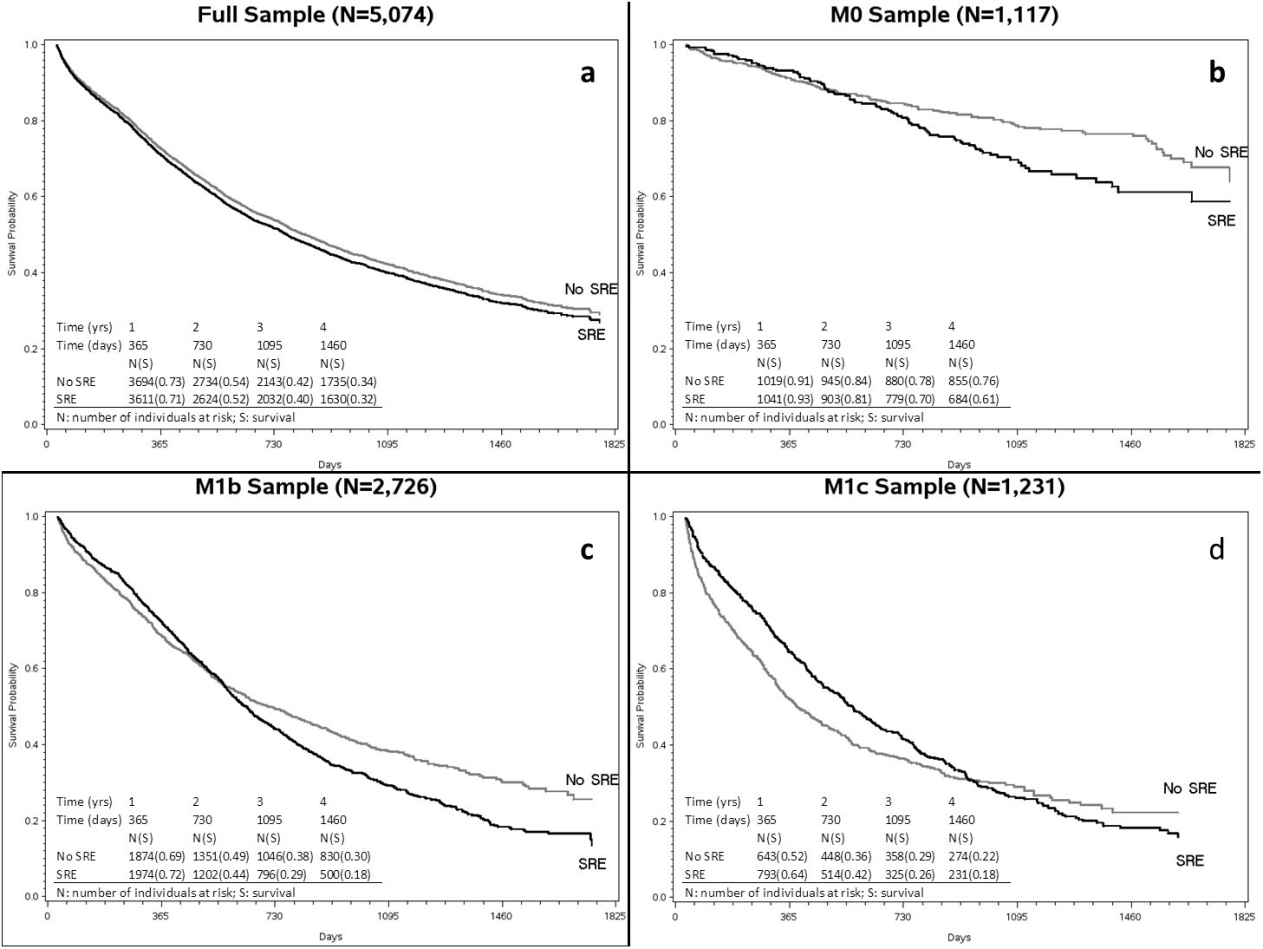
A-D. Direct adjusted survival curves for any SRE (based on Approach 2) in the full sample (Fig 1A), M0 (Fig 1B), M1b (Fig 1C) and M1c (Fig 1D) samples.

**Fig 2:**
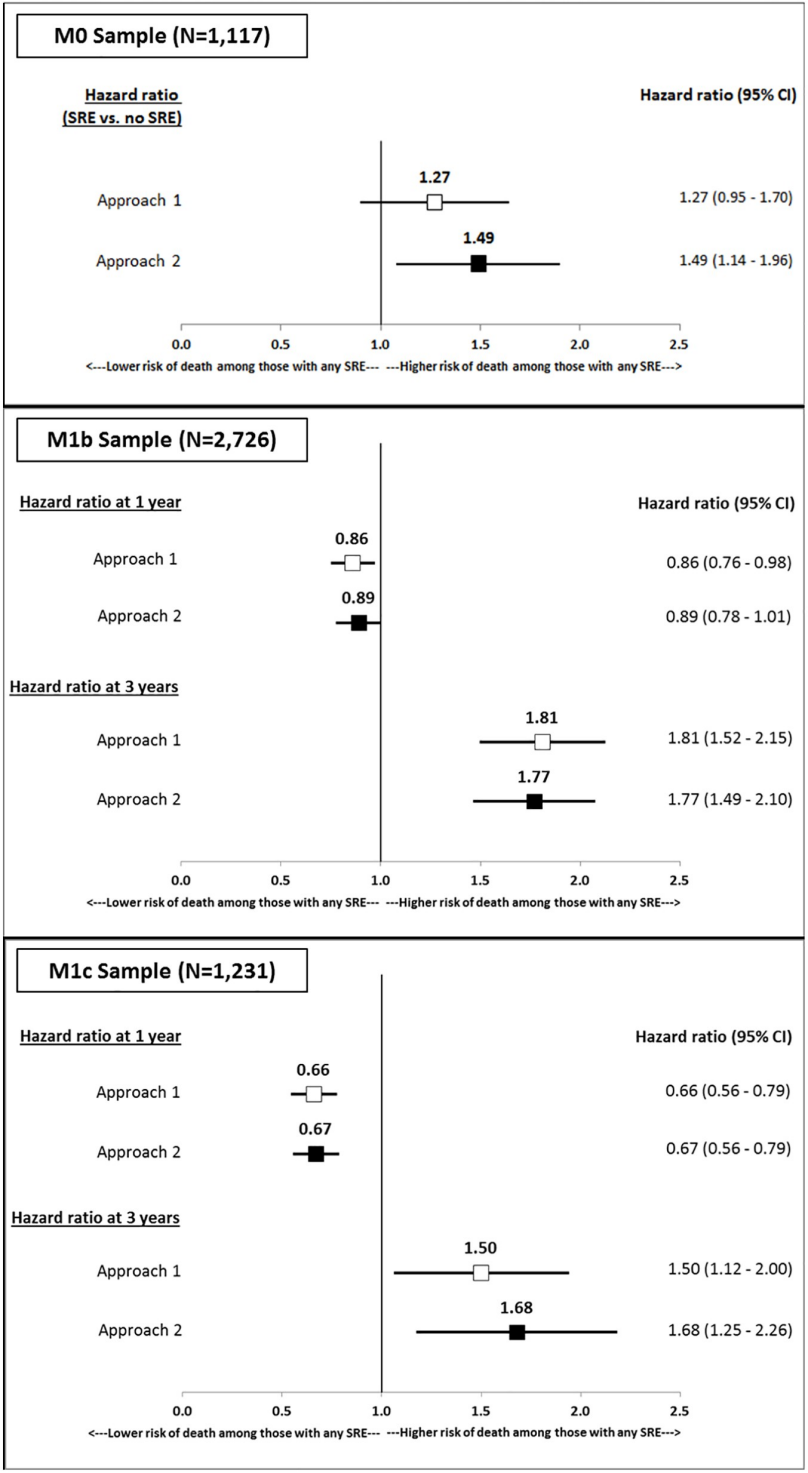
Adjusted hazard ratios (95% CI) associated with SREs for all-cause mortality based on Approaches 1 and 2 in the M0*, M1b** and M1c** samples. The regression models controlled for patient-level demographic factors (age, race, marital status, urban residence), baseline clinical factors (tumor differentiation, Charlson Comorbidity Index, osteoporosis pre-diagnosis, performance status and use of preventive services in the 1 year prior to diagnosis), and other factors including at least 1 month of state buy-in, region of SEER registry and year of diagnosis. *Time invariant hazard ratio reported as the model satisfied proportional hazards assumption. **Hazard ratios reported at 1 year and 3 years post-diagnosis as the models did not satisfy the proportional hazards assumption.

Using Approach 2, there was a statistically significant increased hazard of mortality associated with an SRE in the full sample (Fig 1A) as well as the M0, M1b and M1c subsamples (Fig 1B, 1C and 1D). Conclusions regarding the mortality impact of SREs varied with the definition of RtB embedded in Approaches 1 and 2 (Fig 2). Among M0 patients, the adjusted hazard ratio (AHR) associated with an SRE was 1.27 when using Approach 1 (95% confidence interval: 0.95–1.7) and the AHR was 1.49 when using Approach 2 (95% confidence interval: 1.14–1.96). Among M1b patients, the adjusted hazard ratio (AHR) associated with an SRE varied over time: within one year of diagnosis, it was either below 1 or not statistically significant. At three years following diagnosis, the AHR was 1.81 when using Approach 1 (95% confidence interval: 1.52–2.15) and the AHR was 1.77 when using Approach 2 (95% confidence interval: 1.49–2.1). Among M1c patients, the adjusted hazard ratio (AHR) associated with an SRE also varied over time. The AHR was 0.66 when using Approach 1 (95% confidence interval: 0.56–0.79) and the AHR was 0.67 when using Approach 2 (95% confidence interval: 0.56–0.79). At three years following diagnosis, the AHR was 1.5 when using Approach 1 (95% confidence interval: 1.12–2) and the AHR was 1.68 when using Approach 2 (95% confidence interval: 1.25–2.26).

We investigated the patterns of treatment receipt and survival outcomes in the M1c group in exploratory analyses. We found higher treatment rates among those with SREs vs. no SREs: 24% vs. 12% (p<0.01) for chemotherapy, and 70% vs. 54% (p<0.01) for luteinizing hormone-releasing hormone (LHRH) agonists. In the absence of the appropriate measures for examining disease-specific outcomes (e.g., measures of cancer treatment toxicities such as cardiovascular-specific mortality due to treatment-related toxicities) we examined the broader category of nonPCa-specific mortality. The AHRs for PCa-related mortality were: 0.79 (0.64–0.98) at 1 year; 1.19 (0.92–1.53) at 2 years; and 1.77 (1.10–2.87) at 3 years. The AHRs for nonPCa-related mortality were qualitatively similar: 0.52 (0.38–0.70) at 1 year; 1.03 (0.81–1.30) at 2 years; 2.03 (1.37–3.01) at 3 years.

## Discussion

Claims data linked with registry information provide the opportunity to investigate clinical and mortality impacts of SREs. While claims data provide information regarding receipt of radiation therapy, they do not confirm whether the radiation was delivered to the prostate gland or to the bone. This distinction is critical for identifying radiation to the bone (RtB), one of the events that constitute an SRE. Prior studies [5, 8, 22] have supplemented the claims for radiation therapy by conditioning on a prior claim with a bone metastasis (BM) diagnosis code. We required a prior BM code in the first approach and required a concurrent BM code or a shorter duration of radiation therapy in the second approach. Using both measures, we investigated the mortality impact of SREs in subsamples defined by M stage: M0, M1b, and M1c. This difference in incident diagnosis substage was important because patients with metastasis at diagnosis would not receive radiation for curative intent while the same conclusion could not be drawn for patients with M0 disease.

We found that the conclusion regarding the mortality impact of SREs varied with Approach 1 and Approach 2 among the M0 group but did not vary among the M1 subgroups. The use of Approach 1 did not indicate a statistically significant mortality impact of SREs while use of Approach 2 indicated a statistically significant mortality impact of SREs. It is likely that the RtB instances identified in the M0 group were not limited to receipt of palliative radiation therapy. From Table 3, a minority (i.e., 16%) of the radiation therapy episodes in the M0 sample were less than or equal to 4 weeks in length. This proportion contrasts with the M1b (i.e., 78%) and M1c (i.e., 87%) groups. Among M1b and M1c patients the shorter duration radiation therapy episodes were most likely for palliative purposes: to the bone, and also possibly for other issues like hematuria and local obstructive problems (e.g., obstructive uropathy). Among patients without a BM code, almost half (47.8%) of the radiation therapy episodes in the M0 sample were longer than 8 weeks compared to 21.6% in the M1b group and 11.7% in the M1c group. The higher prevalence of longer duration episodes in M0 is clinically justifiable as radiation may have been for curative intent.

These findings indicate that a significant portion of the radiation episodes in the M0 group will represent radiation to the prostate gland and that it will be particularly important to condition on a BM diagnosis among M0 patients, compared to M1 patients. Prior studies [17, 23] have questioned the reliability of the BM diagnosis code in claims data as an indicator for a diagnosis of BM. Our results reinforce this assertion. A validated measure of BM would identify individuals with non-incident BM in the claims data and could assist with identifying SREs that occur subsequent to BM, particularly among individuals who were not diagnosed with incident BM.

We also found differences between the groups when considering the duration of radiation therapy. In Table 3, more than half of the M1 patients with no BM code during the radiation episode had a radiation episode of 4 weeks or less compared to 1 in 10 M0 patients with no BM code during the radiation episode. Among patients with non-BM coded radiation therapy episodes, the duration requirement served to further isolate potential RtB by focusing on radiation claims that represent a shorter duration of therapy. We expected more episodes of a shorter duration among M1b and M1c patients compared to M0 patients and thus, that more episodes would be picked up from M1b and M1c when applying the duration requirement. Indeed, we find that applying the duration requirement in addition to the BM code requirement picks up 3 additional M1b cases with probable RtB for every additional M0 case and 1.4 M1c cases for every M0 case.

The results regarding the mortality impact of SREs in the M1c group (Fig 1D) warranted further investigation which we accomplished via follow-up exploratory analyses. The seemingly protective effect of SREs within 2 years of diagnosis could be due to selection into treatment SRE components whereby patients receiving treatment SRE components (i.e., radiation, bone surgery) were healthier at diagnosis than patients who did not receive these interventions. We found that the survival curves crossed when examining nonPCa-specific mortality as well as PCa-specific mortality. The crossing of curves in both cases suggests that the eventual increase in mortality among those with SRE was not likely driven by cancer treatment toxicities, or else we would see a crossing only for the nonPCa mortality model but not for the PCa mortality model. Thus, it is likely that the effect at the 3-year mark represents the long-term prognosis of SREs.

There are a few limitations to the study. We do not employ a validated measure of palliative radiation therapy. To the extent that patients without a BM code received longer duration palliative therapy or shorter duration radiation therapy for curative intent, we may have misclassified patients based on the length of radiation therapy. The diagnosis of incident BM that is available from the SEER registry is not validated. While the M1b measure has not been validated, the information about M1 disease in SEER is considered to be reliable thus we expect that our general conclusions regarding the differences between M0 and M1 are robust. Unlike the ICD9 codes used in this study, the new ICD10 set includes codes to identify receipt of palliative radiation although it is too early to determine their reliability. Like ICD9 codes, ICD10 codes may omit other critical features for health services research. In the spirit of a proof-of-concept, the paper shows that differences in measures developed from billing codes can impact study conclusions.

## Conclusion

Studies have documented the clinical and economic burden of skeletal-related events using health care claims data. While these data offer advantages for investigating population-level clinical and cost consequences of SREs, they do not provide the ability to distinguish radiation to the bone from radiation to the prostate. Among patients who do not have an incident diagnosis of metastatic prostate cancer, we found that the definition of radiation to the bone impacted conclusions regarding the mortality impact of SREs. Future studies should validate claims-based measures of radiation to the bone to support studies of SREs among cancer patients, not all of whom will have an incident diagnosis of metastatic disease.
